# Victimization by Caregivers: Prevalence and Risk Factors in Chilean Children and Adolescents

**DOI:** 10.1186/s13034-022-00509-3

**Published:** 2022-09-07

**Authors:** Diego Portilla-Saavedra, Cristián Pinto-Cortez, Cristóbal Guerra, Fabiola Peña Cárdenas

**Affiliations:** 1 Universidad Católica del Norte, Antofagasta, Chile; 2 Escuela de Psicología Y Filosofía, Facultad de Ciencias Sociales y Juridicas, Universidad de Tarapacá, Avenida 18 de Septiembre 2222, Casilla 7-D Arica, Chile; 3 Centro Cielo, Facultad de Ciencias Sociales Y Comunicaciones, Universidad Santo Tomás, Santiago, Chile; 4 Universidad Autónoma de Tamaulipas, Matamoros, México

**Keywords:** Caregiver victimization, Child abuse, Child maltreatment, Child victimization

## Abstract

This study examined the prevalence and risk factors associated to victimization by caregivers in a national large sample of Chilean children and adolescents. 19,687 children and adolescents aged 12–17, selected by random probability sampling of 699 public schools in Chile who were surveyed by trained interviewers. Victimization by Caregivers was evaluated through a module of the Juvenile Victimization Questionnaire (JVQ). The prevalence (12 months) for physical abuse were (12.9%), for emotional abuse (27.9%), neglect (5.3%) and parental interference (3.5%). The results suggest as risk factors, sex, age, migration status, disability, geographical location, and type of school. The findings highlight the urgent need to address the issue of child maltreatment and victimization by caregivers in Chilean society and minimize its impact.

## Introduction

Children and young people are subjected to multiple forms of violence by family members, peers, institutional employees, or strangers, which is manifested in different developmental contexts such as family, school, or community [1, 2]. In this sense, victimization by interpersonal violence suffered by children and young people is configured as a complex phenomenon with a multifactorial origin [3].

Historically, research on child and adolescent victimization has focused on analyzing the most well-known types of violence such as physical or sexual abuse [4], leaving aside other forms of violence that also affect children [5] and their associated risk factors [6, 7].

The limitations of previous investigations have led to the need to reflect on child and adolescent victimization studies. A significant theoretical framework contributing to this analysis is the developmental victimology proposed by Finkelhor, Shattuck [8]. In this model, child victimization has been defined as exposure to all types of interpersonal violence before the age of 18, which adversely affects children and adolescent's developmental growth and psychological well-being [5, 7]. One of the types of victimization considered by developmental victimology is victimization by caregivers [8]. According to Finkelhor, Ormrod [9], caregiver victimization (CV) provides a broader understanding than the classic definition of child abuse. It includes other types of violence by caregivers that have been scarcely addressed in previous studies.

Caregiver victimization in this context has been classified into four types: physical abuse, psychological/emotional abuse, neglect, and parental interference/family abduction [9, 10]. Physical abuse is understood as non-accidental violence and actions caused by caregivers that cause pain to a child or young person and also have the capacity to cause injury or permanent developmental impairment [11]. Regarding psychological/emotional mistreatment Finkelhor and Korbin [11] define it as certain behaviors of caregivers in their relationship with the child such as rejecting, isolating, terrorizing, neglecting, corrupting, or "adultizing" the child. Neglect [12] is conceptualized as the deprivation or non-provision of necessary and socially available resources to the child, potentially creating risks in his or her developmental paths [11]. Finally, parental interference or family abduction refers to the obstruction in the custody of a child or young person, that is, one parent takes away, retains, or hides the child to avoid his or her contact with the other parent [13].

Children and young people who suffer violence at the hands of their caregivers are at the risk of suffering trauma and chronic mental health problems [8]. It has been shown that children in these developmental contexts present depressive [14], anxious [15], post-traumatic [16], substance use [17] and risk behaviors in general [16].

Several studies have been conducted in recent years considering a broad definition of caregiver victimization (CV). For example, in the U.S., in a study with a sample of 4502 children and young people, 4% reported physical abuse, 5.6% reported emotional abuse, 4.7% reported being neglected, and 1.2% reported custodial interference [13]. In Spain, Pereda, Guilera [18] on a sample of 1107 young people aged 12 to 17 years, found that almost one-fifth (18.1%) of the participants had some experience of victimization by caregivers in the 12 months prior to the investigation. In this research, psychological/emotional abuse was the most reported one (13.6%), followed by physical abuse (6.7%) and caregiver neglect and interference (0.9% and 0.4%, respectively) [18].

In Latin America, it has been found that the most frequent types of violence against children and adolescents in caregiving settings are physical abuse, psychological abuse and emotional deprivation [19]. For example, in Mexico, on a sample of 874 children and young people, it was found that 23% of the participants were exposed to CV in the year prior to the research [20], while 16.1% recognized psychological/emotional abuse, 8% physical abuse, 4.2% parental interference/family abduction and 2.4% neglect. Consistent with this study from Mexico, a separate study with a sample of 1068 adolescents reported that 16.5% suffered psychological/emotional maltreatment, 13.8% physical mistreatment, 2.2% parental interference, and 1.4% neglect in the 12 months prior to the study [21].

## Risk factors in caregiver victimization

Studying risk factors for caregiver victimization is relevant because each type of victimization has a specific etiology [6, 22]. In the case of victimization by caregivers, it has been found that sex is a risk factor and women are more likely to suffer violence from their caregivers [23], with physical and psychological abuse being the most frequently reported forms of violence [18, 20, 24]. Regarding age, it has been shown that as age increases, the probability of suffering psychological or emotional abuse by caregivers increases [25, 26]; on the contrary, physical abuse is more frequent in younger children [25]. Similarly, it has been suggested that young children are especially vulnerable to stress caused by abuse since they have fewer biological, psychological, and social resources to cope with these adversities at this stage of development [5]. Moreover, migrant status has also been identified as a risk factor for victimization by caregivers [6], especially when combined with other socio-demographic risks such as socioeconomic status or geographic location [27, 28]. It has been established that low socioeconomic status of migrants is often associated with specific victimizations, such as physical and psychological/emotional abuse [27].

Educational establishment type is associated with the socioeconomic level of the families, and this factor has presented divergent results in the research. For example, it has been highlighted that socioeconomic status, in some cases, acts as an intrinsic stress factor at the family level that would increase the occurrence of victimization by caregivers [29, 30]. However, other studies have found that socioeconomic status is not a significant risk [31], so its influence on the occurrence of CV is not entirely clear.

Results are inconclusive regarding the geographic area of residence, with some research indicating that physical abuse victimization is higher in urban areas than in rural areas [32]. Other studies, on the contrary, indicate that victimizations in rural areas are more frequent and more reported to the police [33]. Children and adolescent victims in rural areas may be more exposed to different forms of child victimization, including victimization by caregivers [34].

Research by Hussey, Chang [6] suggests that ethnicity is also a risk factor for caregiver victimization, specifically for physical abuse and neglect; nonetheless, the research also establishes that young people who identify with an ethnicity are more likely to report mistreatment. However, a combined analysis with other demographic variables shows that ethnic differences in the prevalence of child abuse are mainly due to underlying differences with other socio-demographic risks, such as gender, socio-economic status, schooling, regional origin, and migration status. It is relevant to carry out more studies on risk factors because it has been suggested that certain ethnic groups may be overrepresented in child mistreatment research, generating an associated bias that needs to be considered [35, 36].

Finally, other studies indicate that children or adolescents with disabilities are at a higher risk of suffering physical abuse and neglect [37, 38]. This indicator is especially accurate for children and young people with mental disabilities [39]. The increased risk is also associated with high-stress levels for caregivers in these particular caregiving contexts [40, 41].

## Studies about the caregiver victimization and risk factors in the Chilean context

In Chile, several studies have been implemented to quantify child and adolescent victimization in its various forms [42]; however, only a few of these studies have used probability samples [43, 44]. Additionally, lifetime caregiver victimization has mostly been considered. Only one study considered the prevalence of CV in the last 12 months (prior to the study) in a specific region of northern Chile [45]. Specifically, the study by Pinto-Cortez, Pereda [42] which aimed to examine the state of research on child and adolescent victimization in Chile, found that of the total number of studies reviewed, only 32.1% analyzed caregiver victimization. Additionally, they generally covered the life span and not the twelve months prior to the research. One of the studies comprehensively assessed caregiver victimization, including physical abuse, psychological/emotional abuse, neglect, and parental interference [46]. The obtained results reflect that 52% of the participants suffered psychological/emotional abuse, 27.1% reported physical abuse, 16.3% reported neglect, and 16.4% reported parental interference during their lifetime. These findings are consistent with a later study conducted in Chile in which it was estimated that 52% of the sample suffered from CV during their lifetime [24].

Research on associated risk factors for CV at the national level is scarce and when done has limitations. For example, some analyses have been conducted concerning sociodemographic risk factors, such as sex and age. In this regard, Larrain and Bascuñán [44] found differential aspects; more specifically, they detected that mothers exert more psychological and physical violence toward their daughters. Fathers, on the other hand, perpetrate both mild and severe physical abuse against their sons but more psychological/emotional abuse against their daughters. This study found that 47.7% of participants were physically abused for the first time before the age of 6 years [44]. The study by Pinto Cortez and Venegas [45] using a sample of 706 young people found significant differences between men and women in physical and psychological abuse, the latter form of victimization by caregivers being more frequent in women. However, this study does not consider sex as an associated risk factor.

## The current study

Child abuse and various types of victimization by caregivers are regarded as a severe public health problem and a systematic violation of human rights in Chile [47]. The Chilean state has been promoting public policies to protect children and young people for approximately three decades, especially to guarantee a life free of violence in all their developmental contexts [48]. Therefore, the interrelation between academic research and public management is a fundamental strategy to find a way to address this problem, with concrete data and consequently with remedial measures from the State.

However, regardless of the availability of studies on the subject, research on CV in Chile has focused on assessing victimization during a person's lifetime. Contrary to the most updated approaches to the epidemiology of violence, which argue that it is relevant to assess prevalence/lifetime. Conducting studies considering a shorter period such as prevalence/year would facilitate the task of preventing child abuse more efficiently by the relevant agencies [49]. It is also helpful to add comparisons with representative samples of non-victimized children [13] to do a following of this problem, all of which would be enriched by probability samples with higher representativeness. Also, very few of the studies reviewed here performed specific analyses of risk factors such as age, sex, or presence of some disability for VC, but the results have not been conclusive [29–31, 36].

In Chile, to the best of our knowledge, no study has evaluated victimization by caregivers considering a broader definition than that used in the previous research conducted in Chile, over a more limited period such as 12 months prior to the studies and considering a large national probability sample. Nor do they consider the risk factors associated with caregiver victimization beyond the sex and age variables.

Considering this background, we aimed to examine the prevalence of the four forms of caregiver victimization of children and adolescents in Chile over the 12 months prior to the study and the associated risk factors. The specific objectives are: (1) to estimate the prevalence of victimization by caregivers (physical abuse, psychological/emotional abuse, neglect, parental interference, or family abduction) in the 12 months prior to the study using a national sample, and (2) to determine the associated risk factors (sex, age, educational center, migrant–non-migrant status, ethnic origin, disability, and the geographical area where the child resides).

## Method

### Participants

The study is part of the Initiative called National Survey of Polyvictimization 2017 (ENP) by the Undersecretariat of Crime Prevention of the Ministry of Internal Affairs of Chile and the Council of Childhood and Adolescence. It was conducted simultaneously in the 15 regions of Chile. The target population of the study were all adolescents enrolled between seventh grade of primary education and third grade of secondary education in the directory of enrollment and establishments of the Chilean Ministry of Education during 2016, considering rural and urban areas. The universe corresponds to 2,723,800 adolescents aged 12–19 years residing in Chile at the time of the study [50]. A stratified, tri-stage probability sampling was used according to three criteria: (1) establishment, (2) course/grade, and (3) the number of students in each course.

The sample estimation error was + 0.7 percentage points, with maximum variance and 95% reliability. A post-stratification adjustment was made to weight the sample according to sex, age, educational establishment's administrative unit, and region. The final sample was composed of 18,872 adolescents from 699 educational establishments between 12 and 17 years (M = 14.54, SD = 1.52), of which 49.2% were male and 50.8% female. 96.4% were born in Chile; 5% were children of mothers born in a country other than Chile; 15.4% identified themselves as belonging to indigenous people; and 15.7% reported a permanent physical disability. Table 1 describes the characteristics of the sample used in the study.

**Table 1:** Sociodemographic characteristics of the study sample

	n	%
Sex		
Male	9285	49.2
Female	9587	50.8
Age		
12	3739	9.5
13	1795	19.8
14	3856	20.4
15	3618	19.2
16	3672	19.5
17	2192	11.6
Immigration status		
Non migrant	18,160	96.4
Migrant	684	3.6
School		
Public	8288	43.9
Semi private	9772	51.8
Private	812	4.3
Region		
North	2959	15.7
Center	3896	20.6
South	6262	33.2
Metropolitan	5755	30.5
Ethnicity		
None	15,317	84.6
Indigenous	2787	15.4
Disability		
No	15,900	84.3
Yes	2972	15.7

### Instruments

#### Questionnaire of sociodemographic characteristics

A survey was constructed to ask about specific characteristics of the participants who were asked about: (1) sex of the interviewee; (2) age; (3) migrant status; (4) dependence on the participant's educational establishment; (5) participants' geographic area of residence; (6) self-identification with an indigenous group; (7) permanent disability status.

#### *Juvenile Victimization Questionnaire*—JVQ [10]

An adaptation to the local Chilean context of the Juvenile Victimization Questionnaire (JVQ) created and developed by Hamby, Finkelhor [10] was used. JVQ is a self-report questionnaire designed for adolescents between 12 and 17 years. The estimated application time is 40 min, in which the presence or absence of victimization in the previous year of application or throughout the participant's life is assessed. JVQ assesses the presence or absence of victimization in six fields: victimization by conventional crimes, victimization by caregivers, victimization by peers, sexual victimization, indirect victimization, and electronic victimization. It also provides a continuous score called polyvictimization that corresponds to the sum of all victimization experiences (independent of the type of victimization) throughout the lifetime. For the present study, only the module on victimization by caregivers was used, which is composed of four items, each consisting of one question:

M1. Physical abuse:

Has any adult in your household ever hit, punched, kicked, or physically hurt you in any way?

M2. Psychological/emotional abuse:

Have you ever felt scared or terrible because an adult in your environment called you names, said mean or cruel things to you or said they didn't love you?

M3. Neglect:

When someone suffers from neglect, it means that the adults they live with do not take care of them as they should. It may be because they do not give them enough food, do not take them to a doctor when they are sick, or do not provide a safe place to live.

Have you ever been treated carelessly?

M4. Parental interference/family abduction:

Sometimes families fight about where their children should live. Has a parent or family member ever taken you away, kept you away, or hidden you from your father or mother?

Every item in the questionnaire (e.g., M1) asks for an affirmative (YES) or negative (NO) answer to evaluate the occurrence of this type of victimization during the participant's lifetime. Additionally, a second column asks *how many times this happened to you in the last year?* To evaluate the occurrence of victimization in the last year or a previous year before answering the instrument. JVQ has shown consistently good psychometric properties in its original construction for the U.S. context [4], evidencing Cronbach's alpha of 0.80 for the overall scale, which is considered very good. Cronbach's alpha for the JVQ scales ranges from moderate to weak (common crimes α = 0.61; child maltreatment α = 0.39). The adaptation and validation in the Chilean context of the JVQ also has shown better psychometric properties (JVQ global scale α = 0.85, common crimes α = 0.64, indirect victimization α = 0.64, sexual victimization α = 0.59, peer victimization α = 0.51, caregiver victimization α = 0.50, electronic victimization α = 0.48) [24]. Although the reliability levels are moderate for different modules because these are ordered based on the experts' view and not on the order of the data or the respondents' answers. Another element that may influence the reliability levels is the low number of items. Eventually, other indicators could be considered to evaluate the reliability of the JVQ with different and more robust results [51].

### Procedure

Given that the present study is based on secondary data from the National Polivictimization Survey [52], authorization was requested from the Undersecretariat for Crime Prevention of the Ministry of the Interior of Chile to use the public database. Secondly, the ethical criteria and procedures used to select the sample and implementation of the study were reviewed. Furthermore, the present research followed all principles of the Declaration of Helsinki for research involving human subjects and was reviewed by an ethics committee of the governmental agencies involved in the study. The application of the instruments was carried out between September and December 2017; the sample was obtained through inter-ministerial coordination between the Ministry of the Internal Affairs, the Council for Childhood and Adolescence, and the Ministry of Education. The adolescent participants were accessed through their schools and classrooms, and informed consent and assent were requested from parents and students, respectively, through the educational establishments. The application of the instruments was carried out in the students' classrooms with a trained interviewer and without the teacher's presence. The children and adolescents had to answer the questionnaires silently and then return them to the person in charge. The application took between 40 to 60 min. A total of 19,867 instruments were answered in 699 educational establishments in the 15 regions of Chile, of which 19,684 were eligible for the final analysis as 9.9% of the questionnaires had to be discarded because they were incomplete or had serious legibility problems. Finally, considering that the questionnaires were oriented to inquire about personal experiences that could provoke an emotional reaction of discomfort in the participants, an emotional support protocol was developed for those who would need it. In this context, an e-mail address was provided so that the young people could communicate confidentially with the care mechanisms for victims of violent crime of the Undersecretariat for Crime Prevention of the Chilean Ministry of the Interior, with the aim of providing possible psychological support for any situation that might be affecting them.

### Statistical analysis

The prevalence of victimization of children and adolescents by caregivers was examined using univariate analysis, descriptive analyses were performed to estimate the prevalence/year, and the proportion of children and adolescents who responded affirmatively to suffering each of the forms of abuse consulted in the CV section of the JVQ was calculated. The percentage was calculated in each case, excluding missing data for each response. The mean number of victimizations was calculated as well for the total sample. To evaluate differences according to age and sex we used one-way Anova and Scheffé post hoc test. Multivariate analysis was performed to study the effect of the risk factors associated with each type of VC, specifically logistic regression was used, since this type of analysis predicts the outcome of a categorical variable (VC) in terms of independent or predictor variables. In this study, the independent variables used were sex, age, educational establishment branch, the origin of the children or adolescent (migrant–non-migrant), ethnic origin, disability, and geographic area where the children or adolescent resides. Logistic regression was used to model the probability of occurrence of the event (VC) as a function of other factors [53]. The statistical package SPSS version 26.0 was used to perform the statistical analyses.

## Results

In the present study, psychological/emotional abuse was reported by 27.9% of the participants, physical abuse by 12.9%, neglect by 5.3%, and parental interference by 3.5% at the 12 months previous to the study (See Table 2).

**Table 2:** Prevalence (12 months) of victimization by caregivers in Chilean children and adolescents (95% CI)

	12 years old	13 years old	14 years old	15 years old	16 years old	17 years old	Total
Total							
Physical abuse	12.7 (11.1–14.3)	13.5 (12.3–14.6)	14.9 (13.7.16,1)	13.7 (12.5–14.8)	11.2 (10.2–12.3)	10.4 (9.0–11.7)	12.9 (12.4–13.4)
Emotional abuse	24.0 (21.9–26.1)	25.5 (24.0–26.9)	28.4 (26.9–29.9)	29.7 (28.2–31.3)	29.4 (27.9–31.0)	29.2 (27.2–31.1)	27.9 (27.3–28.6)
Neglect	4.1 (3.1–5.1)	4.4 (3.7–5.1)	5.2 (4.5–6-0)	5.2 (4.5–6.0)	5.7 (4.9–6.5)	6.4 (5.3–7.5)	5.3 (4.8–5.5)
Parental interference	3.4 (2.5–4.2)	3.5 (2.9–4.1)	4.3 (3.7–5.0)	3.4 (2.8–4.0)	3.0 (2.4–3.6)	2.2 (1.6–2.9)	3.5 (3.1–3.7)
Number of victimizations*	0.44 (0.73)	0.47 (0.76)	0.53 (0.82)	0.52 (0.80)	0.49 (0.76)	0.48 (0.76)	0.50 (0.78)
Male							
Physical abuse	11.3 (9.0–13.6)	12.1 (10.5–13.6)	11.1 (9.7–12.6)	11.6(10.1–13.2)	10.0 (8.6–11.5)	8.8 (7.1–10.5)	10.9 (10.3–11.6)
Emotional abuse	16.9 (14,2–19.6)	16.9 (15.0–18.7)	17.8 (16.0–19.6)	20.0 (18.0–21.9)	18.5 (16.6–20.3)	19.2 (16.8–21.5)	18.3 (17.4–19.1
Neglect	3.3 (2.0–4.6)	3.5 (2.6–4.4)	3.9 (3.0–4.8)	4.0 (3.1–4.9)	4.3 (3.4–5.3)	4.5 (3.2–5.7)	4.0 (3.5–4.4)
Parental interference	2.5 (1.3–3.6)	2.8 (2.0–3.6)	3.7 (2.8–4.6)	2.6 (1.8–3.4)	2.3 (1.6–3.1)	2.1 (1.2–2.9)	2.7 (2.4–3.1)
Number of victimizations*	0.34 (0.62)	0.35 (0.69)	0.37 (0.70)	0.38 (0.68)	0.35 (0.67)	0.35 (0.67)	0.36 (0.67)
Female							
Physical abuse	13.9 (11.6–16.1)	14.7 (13.1–16.4)	18.7 (16.9–20.5)	15.6 (13.9–17.3)	12.4 (10.8–14.0)	12.1 (10.0–14.1)	14.9 (14.1–15.6)
Emotional abuse	29.8 (26.8–32.8)	33.3 (31.1–35.5)	39.1 (36.9–41.4)	39.2 (36.9–41.5)	40.3 (38.0–42.7)	40.1 (37.0–43.2)	37.4 (36–4-38–5)
Neglect	4.8 (3.4–6.2)	5.2 (4.2–6.3)	6.7 (5.5–7.8)	6.4 (5.2–7.5)	7.1 (5.9–8.3)	8.5 (6.7–10.2)	6.4 (5.9–6.9)
Parental interference	4.1 (2.8–5.4)	4.1 (3.2–5.0)	5.1 (4.1–6.1)	4.2 (33–5.2)	3.7 (2.8–4.5)	1.5 (1.5–3.4)	5.1 (3.6–4.5)
Number of victimizations*	0.53 (0.80)	0.57 (0.82)	0.70 (0.90)	0.65 (0.88)	0.63 (0.81)	0.63 (0.82)	0.63 (0.84)

*Number of victimizations is referred to the number of different types of victimization by caregivers suffered in the last year: Mean (SD). In all cases range was between 0 and 4

In addition, the results show that 65.3% (n = 11,394) of the participants had not suffered any of the four types of victimization by caregivers in the last year while 34.7% (n = 6065) had suffered at least one of the four types of victimization. Specifically, 22.7% (n = 3961) indicated having suffered one of the four types of violence; 9.7% (n = 1689) reported having suffered two; 2.0% (n = 356) reported having suffered three of them; and 0.3% (n = 59) had suffered all four. The mean number of victimizations by caregivers was 0.50 (SD = 0.78) in the total sample. Please see Table 2 to see this information divided by age and sex. Females suffered an average of 0.63 victimizations in the last year (SD = 0.84) and males 0.36 (SD = 68). This difference is statistically significant (t_(17457)_ = 23,120, p < 0.01). There were differences across age as well (F_(5, 17453)_ = 4,662; p < 0.01). As can be seen in Table 2, the highest mean number of victimizations across last year was in 14 and 15 years old. In fact, post hoc analysis with Scheffé test show that this difference is statistically significant when participants 14 years old are compared with participants 12 years old (mean difference = 0.09; SE = 0.02; p < 0.05) and 13 years old (mean difference = 0.06; SE = 0.02; p < 0.05). In the same way, there is a statistically significant difference in the score of participants of 15 years old compared to the ones of 12 years old (mean difference = 0.08; SE = 0.02; p < 0.01).

Logistic regression analysis was used to evaluate whether sex, age, immigration status, socio-economic level on educational establishment, area of residence, ethnic membership, and disability status increased the probability of suffering each of the four types of violence evaluated (Table 3 shows the OR the last 12 months).

**Table 3:** Logistic regression coefficients (and standard error) of the factors associated with the forms of CV

	PA	EA	NEG	PI
Sex	.711***	0.367***	.624***	.648***
(cat.ref: male)	(.046)	(.036)	(.070)	(.085)
Age	.949***	1.066 ***	1.100***	.917**
	(.015)	(.011)	(.023)	(.028)
Immigration status	.888	1.083	.953	.667*
(cat.ref.: Non migrant)	(.120)	(.098)	(.183)	(.193)
School				
(cat.ref.:private) Semi private	.914	1.220*	1.100	1.358
	(.106)	(.088)	(.183)	(.235)
Public	.770*	1.060	1.290	1.346
	(.108)	(.089)	(.184)	(.237)
Region				
(cat.ref.: Metropolitan) North	.897	.839**	.999	.733*
	(.070)	(.054)	(.104)	(.130)
Center	.857*	.882*	.896	.731**
	(.054)	(.049)	(.098)	(.120)
South	.867*	.828***	.837	.722**
	(.078)	(.044)	(.089)	(.106)
Ethnicity	.878*	.935	.900	.847
(cat.ref.: have no ethnicity)	(.062)	(.049)	(.094)	(.112)
Disability	.716***	.677***	.535***	.482***
(cat.ref: have no physical and/or mobility impairment)	(.059)	(.047)	(.081)	(.096)

*PA*  physical abuse, *EA* psychological/emotional abuse, *NEG*  neglect, *PI* parental interference/family abduction\

**p < 0,01; *p < ,05;

The probability of having suffered physical abuse during the past year is higher in females (OR: 0.711, 95%CI [0.650, 0.777]), in younger adolescents (OR: 0.949, 95%CI [0.922, 0.977]), in private schools in comparison to public school (OR: 0.770, 95%CI [0.623, 0.952]), in the metropolitan region than central zone (OR: 0.857, 95%CI [0.755, 0.973]) and south (OR: 0.867, 95%CI [0.775, 0.971]), in ethnic groups (OR: 0.878, 95%CI [0.777, 0.991]), and in adolescents with disability (OR: 0.716, 95%CI [0.638, 0.804,]).

Psychological abuse also was more likely to happen among females (OR: 0.367, 95%CI [0.342, 0.394]), among older adolescents (OR: 1.066, 95%CI 1.043, 1.091]), in semi-private schools in comparison to private schools (OR: 1.220, 95%CI [1.027, 1.449]), in the metropolitan region than in the north (OR: 0.839, 95%CI [0.754, 0.934]), center (OR: 0.882, 95%CI [0.801, 0.970]), or south (OR: 0.828, 95%CI [0.759, 0.903]) and in adolescents with some disability (OR: 0.677, 95%CI [0.617, 0.741]).

Neglect also occurred more frequently among females (OR: 0.624, 95%CI [0.544, 0.716]), among older adolescents (OR: 1.100, 95%CI 1.052, 1.150]), and among adolescents with disabilities (OR: 0.535, 95%CI [0.456, 0.627]).

Finally, parental interference is more prevalent too for females (OR: 0.648, 95%CI [0.548, 0.766]), for younger adolescents (OR: 0.917, 95%CI [0.869, 0.969]), for migrant population (OR: 0.667, 95%CI [0.458, 0.974]), in the metropolitan region compared to in northern (OR: 0.733, 95%CI [0.569, 0.946]), central (OR: 0.731, 95%CI [0.578, 0.924]) or southern (OR: 0.722, 95%CI [0.587, 0.888]) regions, and in adolescents with disabilities (OR: 0.482, 95%CI [0.399, 0.582]).

## Discussion

The aim of this research was to identify prevalence/year and risk factors associated with victimization by caregivers in a national sample of Chilean children and adolescents. The findings show that the prevalence of victimization by caregivers was 34.7%, which is a higher figure if we consider other studies performed in developed countries with a similar methodology and with the same measurement instrument [10, 13], for instance in Spain [18], the United States [9] or the United Kingdom [49].

The same tendency is repeated when the types of victimization by caregivers are analyzed in detail. In the present study, psychological/emotional abuse was reported by 27.9% of the participants, physical abuse by 12.9%, neglect by 5.3%, and parental interference by 3.5%; all these figures surpass the values reported in research from some developed countries [9, 18, 49] (Table 2 shows the prevalence (12 months) of victimization by caregivers). Comparing these results with those from other Latin American countries, such as Mexico, where two studies have been carried out using the same approach [20, 21], the figures are divergent. For example, in the case of physical abuse, the data from the Chilean study are higher than those of one of the Mexican studies [21] but lower than those of the second study [20]. It should be emphasized that both studies were conducted in two different regions of Mexico. Psychological/emotional abuse in the present study was significantly higher than in both Mexican studies, as was neglect. Regarding the prevalence of parental interference reported by the Chilean sample, these are slightly higher than in the study by Méndez-López and Pereda [21] but lower than in the study by Peña Cárdenas, Pinto Cortez [20].

Comparing the figures obtained with previous Chilean studies on caregiver victimization, we found different results. For example, the study of UNICEF [50] reported a much higher prevalence of victimization by caregivers, for example, 65.1% of physical violence by caregivers and 56.1% of psychological violence. Although these data could be viewed positively, as they could be interpreted as a decrease in victimization by caregivers in Chile, they should be analyzed with caution. In particular, the UNICEF study assessed only two forms of victimization by caregivers, physical and psychological violence. Each type of violence was assessed with three and four sub-items, respectively.

Some items were confusing, and it was not entirely clear whether they belonged to other types of victimization; an example is "Threatened them with using or has used a gun, knife or other weapons against them," where the assessment of two types of victimization seems to be included. In contrast, items of mild physical violence "Slapped him/her or threw things that could hurt them" and in the same construct "Slapped or spanked them" are included. These are very difficult items to differentiate if they are assessing the same thing or not.

There are also other issues to care for, such as the characteristics (only eighth-grade students were evaluated) and the size of the sample (N = 1555), which could have impacted the results. Notwithstanding the possible methodological differences and their possible influence on the results, both our study as well as the study of UNICEF [50] highlight the large proportion of Chilean children and adolescents affected by caregiver victimization. Given that Chile is currently part of the OECD, where the main developed and developing countries participate, this is relevant, as Chile aspires to position itself as a country that endeavors to reduce opportunity gaps, paying particular attention to the development of effective public policies for the care, education and child protection [54].

However, and in light of the results, it is necessary to go deeper into these issues and analyze the extent to which public policies for the protection of children are producing results or not. It is imperative to consider whether these high figures (in comparison with other countries) are due to aspects related to prevention, such as greater visibility of abuse, as shown in official reports [55]. Undoubtedly, by itself, this would be a positive development.

A worrying statistic was the prevalence of emotional/psychological abuse in the national sample, which is the highest type of violence committed compared to the other types of victimization by caregivers. These results align with what has been previously stated regarding violence against children as a complex social phenomenon that persists in various forms in Latin American countries [3, 19, 46] and specifically in Chile [24, 45]. These findings are directly related to preliminary national studies with non-probabilistic samples that report psychological/emotional abuse as the most recurrent in the Chilean population in similar age ranges, although at different times [45, 56]. These results could be related to the current barriers preventing physical abuse of children and adolescents, mainly because of the physical repercussions it generates, which are visible and therefore alarm people who interact with children at an early stage, but psychological abuse continues to be invisible and naturalized in the name of punishment or parenting practices.

One crucial factor in the prevalence of victimization by caregivers is gender. Studies by Fite, Williford [23] and Hunnicutt [57] suggest that women suffer different types of violence because we live in modern societies that are structurally sexist or essentially patriarchal, factors that are firmly rooted within families. These findings are consistent with those of the present study, in which the female sex is found to be a risk factor for all types of victimization by caregivers.

In relation whit age the results showed the highest mean score of victimization by caregivers at 14 and 15 years old. This is coherent with the developmental perspective that highlights that puberty, and the associated changes may be a source of conflict between child and caregiver that could explain the highest scores in 14 and 15 years old [58, 59]. This idea should be explored in future studies since analysing each form of victimization by caregivers separately, the regression analysis shows a differentiator role of age in the same way as a previous study [25] that points out that younger children are more vulnerable to physical abuse, while, as age increases, they suffer more from emotional/psychological abuse or neglect or even more of the types of victimization by caregivers. Coinciding with the findings of this study and because of the fact that in the pre-adolescent stage they develop better physical capacities that would allow them to stop or defend themselves from physical abuse [20], and out of fear too, parents tend to use psychological violence. The vulnerability to the combined action of multiple types of violence could be explained by the scarce parental tools that caregivers have, which are essential to accompany the process of childhood, preadolescence, and adolescence, in which different changes occur at the biological, cognitive, and emotional levels [60].

Regarding the question of whether the type of educational establishment, private, public, or semi-private, constituted a risk of victimization by caregivers, a statistical significance was found between private establishments and the probability of occurrence of physical maltreatment and semi-private establishments as a risk factor for psychological maltreatment. In Chile, the type of educational establishment has generally been linked to the socioeconomic level of the families. That is, families that keep their children in private schools may have higher incomes, compared to families with a medium or low socioeconomic level, who generally choose to enroll in semi-private or public schools, respectively. In this sense, our findings are contrary to the literature that has related a higher frequency of victimization by caregivers at lower socioeconomic levels [61]. In this area, a plausible explanation is in line with some research that suggests that physical abuse is more frequent in wealthier families, given that in these families there are greater expectations of their children, and therefore greater pressure is exerted from an authoritarian parenting style [62]. However, in the case of psychological mistreatment in semi-private schools, this could be explained by the family stress model [63], which indicates that insecurities or economic instability exert pressure on families, leading to increased stress and mental health difficulties for caregivers, which could be related to a greater probability of psychological mistreatment [64]. Economic instability and insecurity is a deep-rooted characteristic of Chilean middle-class families, given that they must deal with the structural inequalities at the socioeconomic level prevailing in the country, so a higher level of indebtedness is to be expected in order to maintain their social status, in key areas such as education, health and food [65], elements that could increase family stress.

As for situational and community factors, significantly, children and young people living in the large urban metropolitan area were more vulnerable to physical abuse, psychological abuse, and parental interference. Our results are consistent with previous studies that point to urbanity as a risk factor [33]; however, they differ from studies that argue that rurality is a risk factor for victimization by caregivers [34]. The high frequency of interpersonal violence in urban Latin American areas, and especially within families and caregiving relationships, could be related to (1) macro-social factors such as social inequality due to increased wealth and poverty, the paradox of higher educational level but lower employment opportunities, increased expectations and the impossibility of meeting them, changes in the family and the loss of religion/spirituality/social support in people's lives; (2) meso-social the increase of density in poor areas and urban segregation; (3) micro-social such as alcohol consumption and the difficulty of verbal expression of people's feelings [66].

Ethnic group identification has been considered as a risk factor for victimization by caregivers [6]. Along these lines, we found in the present study that belonging to an ethnic group is a risk factor for physical abuse. Nevertheless, these results should be considered with caution as it is highly probable that ethnicity interacts with other underlying risk factors such as socio-economic status or migrant status, a situation that has been shown in previous studies as well [35, 36, 67] and needs to be explored in depth in the future, particularly in the Latin American context where it has been scarcely explored.

The variable of migrant status alone was associated with parental interference as a risk factor in the present study. Moreover, our results align with previous research, indicating that this form of victimization could even involve relocating a child to another country, region, or state [68].

Regarding disability, the findings indicate that people with disabilities are more likely to suffer the four types of victimization by caregivers. These results coincide with a broader line of research that supports the relationship between disability, physical abuse [69], and psychological abuse [70] and presents new data on parental interference, particularly in the Chilean context, and can be considered in future studies.

This research has followed a rigorous procedure from design to analysis. Still, it is important to point out some limitations. It represents only Chilean children and adolescents in school, even though Chile has a high rate of home schooling among children and adolescents. It is known that a considerable proportion of Chilean children and adolescents are not enrolled in school and, therefore, it could be expected that these adolescents are exposed to multiple forms of violence, including victimization by caregivers. Consequently, the non-inclusion of this population is a limitation of the study. The cross-sectional nature of the design means that no causal association can be attributed to the events studied. It also should be stressed that the data are subject to the limitations of any study based on self-reporting.

## Practical implications

The findings of the present study may have important practical implications for the prevention of victimization by caregivers (and not only physical or psychological abuse). The results can be transferred in a formative way to professionals working in childcare and families. Although the analysis of the figures in this study can help families become aware of the seriousness of the problem, it is also important to provide families with parenting strategies that substitute physical violence. For this reason, the influence that the family has on the healthy development of children and adolescents is paramount.

Regarding the results on risk factors, these can be useful for the authorities implementing plans and programs to prevent child abuse, for example, agencies such as the National Service for Children in Chile, the Ministry of Education, and the Ministry of Social Development of Chile. The resources could be optimized by carrying out prevention focused on the vulnerable groups identified in this research. It is recommended to provide information to guide intervention programs at the family and individual levels to reduce the likelihood of future problems at the individual level, which could also be used as a preventive element. The results of this research suggest it may be useful for those programs to consider age, gender, ethnicity, migrant status, and socioeconomic status as they are associated with different types of caregiver violence and show be addressed in a particular way. Future research and public policy should work on those types of programs.

## Conclusion

In summary, the present study shows that victimization by caregivers affects a significant percentage of Chilean children and adolescents, with psychological/emotional abuse being the most prevalent type of violence. The findings show that psychosocial factors are present in child and adolescent abuse. Practically speaking, these factors could be considered relevant as a scientific basis for the formulation of public policies, emphasizing prevention and intervention regarding these specific forms of victimization.

## Data Availability

This data is part of a secondary study. The data are available for the universities from Chile transferred from the Government of Chile. But the data are anonymous, and it is not possible to identify the participants.
